# Novel frontiers in gene therapy: In vivo gene editing

**DOI:** 10.1002/hem3.25

**Published:** 2024-01-28

**Authors:** Francesca Vinchi

**Affiliations:** ^1^ Iron Research Laboratory, Lindsley F. Kimball Research Institute New York Blood Center New York City New York USA; ^2^ Department of Pathology and Laboratory Medicine Weill Cornell Medicine New York USA

Human gene therapy aims at modifying or manipulating the expression of a gene within cells for therapeutic purposes, with the final goal to cure diseases, including blood and solid cancers, as well as genetic and infectious diseases. Nowadays, through gene therapies and gene‐editing approaches, it is possible to replace a disease‐causing gene with a healthy gene copy, inactivate a disease‐causing gene that is not functioning properly, or introduce a new or modified gene to help treat a disease.

Advances in gene‐editing and cellular therapies are revolutionizing care for patients with hematologic diseases, finally offering promising new therapeutic options for their underlying conditions.

β‐Thalassemia and sickle cell disease are the most common monogenic diseases in the world. Patients with these blood disorders commonly develop anemia and severe chronic pain, which tremendously affect their quality of life. These diseases are potentially curable through allogeneic hematopoietic stem cell (HSC) transplantation or autologous HSC transplantation after genetic modification.^1^, ^2^, ^3^ Autologous gene therapy potentially offers a universal cure that overcomes many limitations of allogeneic HSC transplantation, including the lack of available donors, graft‐versus‐host disease, and graft rejection.^1^, ^2^, ^3^

Significant progress in gene therapy for hemoglobinopathies has been made over the last decade, with multiple clinical trials currently investigating both gene addition and gene‐editing strategies.^3^ Recently, a new milestone was reached in the field of gene therapy, with the improvement of gene‐editing tools. A team of investigators in Philadelphia, led by Hamideh Parhiz, Stefano Rivella, and Drew Weissman, developed a proof‐of‐concept model for delivering gene‐editing tools to the bone marrow to treat blood disorders, allowing for in vivo cellular reprogramming and providing a new and potentially safer way of controlling HSCs fate by correcting genetic defects.^4^ If translated into the clinic, this approach could expand access and reduce the cost of gene therapies for blood disorders.

This remarkable study provides us with a revolutionary methodology to perform gene editing with unprecedented advantages. Until today, gene therapy for hematologic diseases implied the collection of HSCs from patients, followed by their in vitro expansion and the modification of the target gene through lentiviral transduction or gene editing. After collection, the patient's HSCs are modified to correct the disease‐causing gene and then transplanted back after myeloablative chemotherapy.^1^, ^3^ Myeloablation, which often causes toxicity, is required to eliminate the mutated HSCs in the bone marrow and makes space for the transduced or edited functional cells.^2^

The research study published in *Science* by Breda et al. describes and shows the efficacy of a novel and so‐far unforeseen approach to genetically modify HSCs in vivo, without the need for donor cells or any prior in vitro manipulation. This methodology takes advantage of messenger RNA (mRNA), used to correct a disease, encapsulated in lipid nanoparticles (LNPs).^4^, ^5^, ^6^, ^7^, ^8^ LNPs are highly effective at packaging and delivering mRNA to cells and were widely used in 2020 when the LNP–mRNA platform was applied to generate two major coronavirus disease 2019 (COVID‐19) vaccines.^5^, ^8^

While for COVID‐19 vaccines the LNP–mRNA construct did not target specific cells or organs within the body, Breda et al. took advantage of this technology to target long‐ and short‐term HSCs. To achieve cell‐specific delivery the surface of the LNPs was decorated with antibodies that recognize c‐Kit (CD117), a receptor expressed on the plasma membrane of HSCs.^4^ Applying this improved LNP technology, the researchers not only successfully proved that this innovative strategy genetically alters HSCs and corrects a disease mutation in vitro but also showed that this method allows direct targeting and durable gene editing of stem cells in vivo after a single systemic injection of modified LNPs, paving the way for an entirely novel approach of gene editing and gene therapy.^4^

Last but not least, the study shows the broader applicability of the methodology for additional scopes, including the development of a nongenotoxic myeloablative conditioning strategy. Breda and co‐authors showed that HSCs can be depleted through LNP‐mediated delivery of the mRNA for the proapoptotic p53‐upregulated modulator of apoptosis PUMA, capable of triggering stem cell death.^4^ In such a way, the targeted LNP–mRNA system offers an optimal strategy for stem cell depletion in preparation for stem cell transplant procedures, with the exceptional advantage of avoiding the acute and chronic toxicities associated with the current chemotherapy or radiation conditioning regimens. Although this conditioning approach requires refinement to restrict LNP tropism and limit gene expression in unintended cells, it has the potential to replace current myeloablative regimens.

These pioneering studies prove that the potential of these targeted mRNA‐based therapeutics goes far beyond the correction of genetic mutations for the cure of monogenic disorders, including nonmalignant hematopoietic disorders (hemoglobinopathies, congenital anemias or thrombocytopenias, and immunodeficiencies) and nonhematopoietic diseases (cystic fibrosis, metabolic disorders, and myopathies), and extends to myeloablation for stem cell transplantation procedure as well as the modification of stem cell physiology for reprogramming purposes (Figure 1).

**Figure 1:**
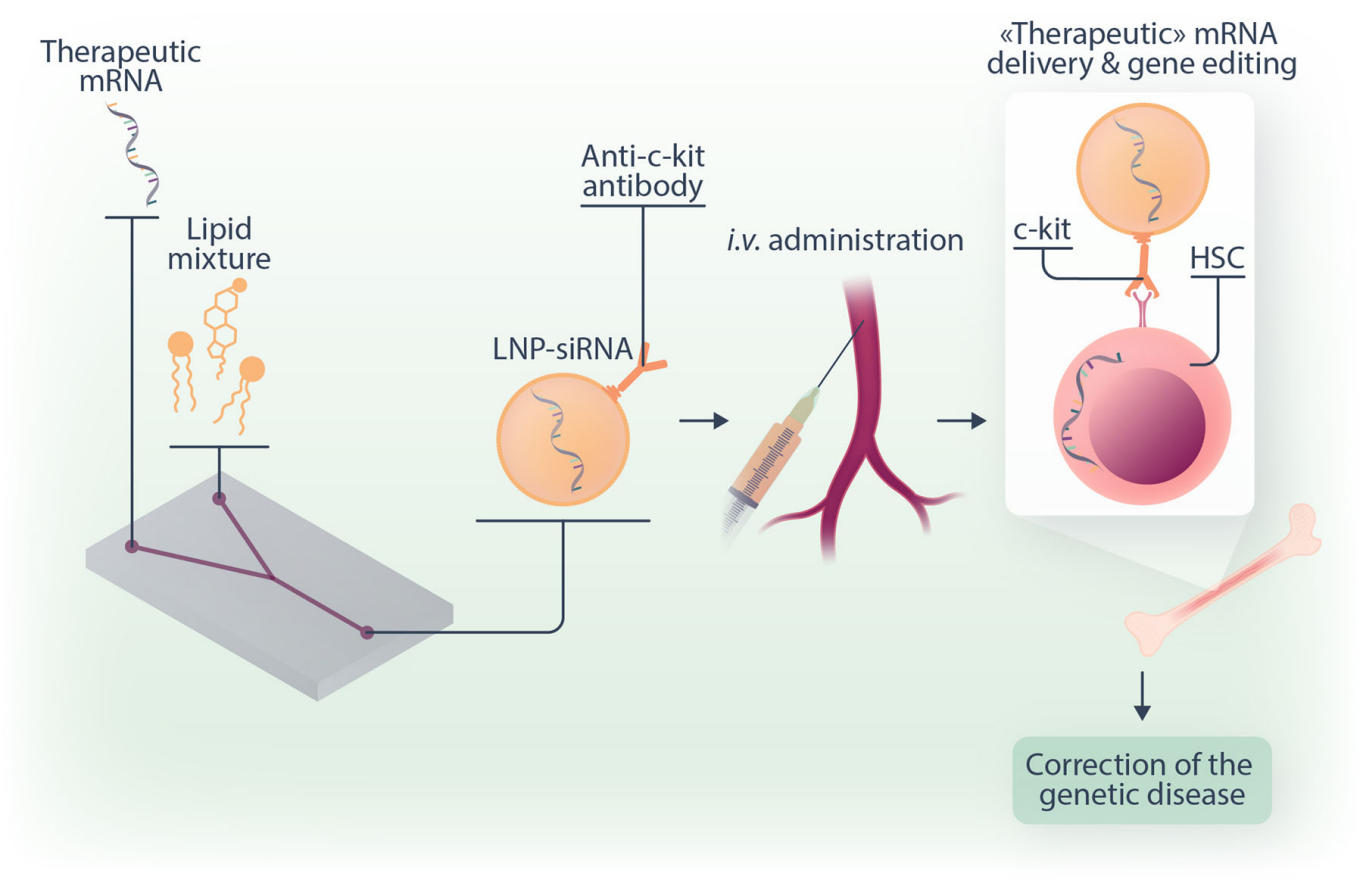
Gene therapy via targeted LNP–mRNA. LNP obtained by mixing different types of lipids are engineered in a way to encapsulate the “therapeutic” mRNA of interest and decorated with antibodies against the stem cell receptor c‐Kit (CD117). Following intravenous administration, the LNP–mRNA technology targets HSCs in the bone marrow, facilitating in vivo gene editing and the correction of the underlying genetic disease. HSC, hematopoietic stem cell; LNP, lipid nanoparticle; mRNA, messenger RNA; siRNA, small interfering RNA.

## AUTHOR CONTRIBUTIONS

Francesca Vinchi conceived and wrote the HemaTopic.

## CONFLICT OF INTEREST STATEMENT

Dr. Vinchi is a member of the advisory board of Silence Therapeutics, a consultant for RallyBio. Dr. Vinchi receives funding from CSL Vifor, Silence Therapeutics, and PharmaNutra.

## FUNDING

This research received no funding.

## Data Availability

Data sharing does not apply to this article as no new data were created or analyzed in this study.
